# Lipid Profiling Reveals Browning Heterogeneity of White Adipose Tissue by Β3-Adrenergic Stimulation

**DOI:** 10.3390/biom9090444

**Published:** 2019-09-03

**Authors:** Ping He, Biyu Hou, Yanliang Li, Chunyang Xu, Peng Ma, Sin Man Lam, Victoria Gil, Xinyu Yang, Xiuying Yang, Li Zhang, Guanghou Shui, Junke Song, Guifen Qiang, Chong Wee Liew, Guanhua Du

**Affiliations:** 1 State Key Laboratory of Bioactive Substances and Functions of Natural Medicines, Institute of Materia Medica, Chinese Academy of Medical Sciences and Peking Union Medical College and Beijing Key Laboratory of Drug Target and Screening Research, Beijing 100050, China; 2 Department of Physiology and Biophysics, College of Medicine, University of Illinois at Chicago, Chicago, IL 60612, USA; 3 State Key Laboratory of Molecular Developmental Biology, Institute of Genetics and Developmental Biology, Chinese Academy of Sciences, Beijing 100101, China; 4 College of Pharmacy, Guangdong Medical University, Dongguan 523808, China

**Keywords:** lipid profiling, white adipose tissue, browning heterogeneity, visceral adipose tissue, subcutaneous adipose tissue

## Abstract

Background: White adipose tissue (WAT) browning confers beneficial effects on metabolic diseases. However, visceral adipose tissue (VAT) is not as susceptible to browning as subcutaneous adipose tissue (SAT). Aim: Interpreting the heterogeneity of VAT and SAT in brown remodeling and provide promising lipid targets to promote WAT browning. Methods: We first investigated the effects of β3-adrenergic stimulation by CL316,243 on systemic metabolism. Then, high-coverage targeted lipidomics approach with multiple reaction monitoring (MRM) was utilized to provide extensive detection of lipid metabolites in VAT and SAT. Results: CL316,243 notably ameliorated the systemic metabolism and induced brown remodeling of SAT but browning resistance of VAT. Comprehensive lipidomics analysis revealed browning heterogeneity of VAT and SAT with more dramatic alteration of lipid classes and species in VAT rather than SAT, though VAT is resistant to browning. Adrenergic stimulation differentially affected glycerides content in VAT and SAT and boosted the abundance of more glycerophospholipids species in VAT than in SAT. Besides, CL316,243 increased sphingolipids in VAT without changes in SAT, meanwhile, elevated cardiolipin species more prominently in VAT than in SAT. Conclusions: We demonstrated the browning heterogeneity of WAT and identified potential lipid biomarkers which may provide lipid targets for overcoming VAT browning resistance.

## 1. Introduction

White adipose tissue (WAT) and brown adipose tissue (BAT), two general types of adipose tissue, are critical for maintenance of metabolic homeostasis. As unilocular white adipocytes, WAT is typically classified as subcutaneous adipose tissue (SAT) and visceral adipose tissue (VAT) [1]. Accumulation of VAT is strongly associated with metabolic diseases and cardiovascular risk, however, increase in SAT confers little or no risk and may even be protective, indicative of WAT heterogeneity [2,3,4,5]. BAT, which contains multilocular fat droplets and abundant mitochondria, is specialized for energy expenditure through non-shivering thermogenesis led by uncoupling protein 1 (UCP1) [6]. It has been reported that the dynamic turnover of adipose tissue in its mass and even type through expansion and remodeling may happen according to the body’s energy status [7,8]. 

Brown remodeling of WAT, the emergence of brown-like adipocytes in WAT, switches WAT into an energy-disposal depot instead of an energy storage site and has become a promising strategy against obesity [9]. SAT is highly susceptible to browning, whereas VAT shows resistance in brown transdifferentiation [10]. To date, the mechanisms are not yet well understood. Therefore, to promote WAT browning, it is critical to further elucidate the browning heterogeneity of SAT and VAT.

Lipids are important biomolecules and serve numerous vital biological functions, which can be identified and quantified by lipidomics including lipid classes and molecular species [11]. Over the past ten years, lipidomics analysis has been performed to investigate the effects of diet, exercise, or cold exposure on WAT, especially SAT, and BAT, and identified several specific thermogenic biomarkers in lipids, such as cardiolipin and ceramide [12,13,14,15,16,17]. However, few studies paid attention to VAT, the important pathogenic adipose tissue. In particular, the browning differences between SAT and VAT in the lipidomics signature have not been investigated yet, let alone the lipid biomarkers. Given that adipose tissue is innervated and activation of the sympathetic nervous system dwindles fat stores primarily attributing to thermogenic β3-adrenergic receptor [18], understanding the differences in lipidomics adaptation between SAT and VAT in CL316,243-induced brown remodeling may provide improved insights into the heterogenous mechanism of WAT browning, and is a prerequisite for overcoming VAT browning resistance.

In the present study, we firstly examined the pharmacodynamics effects of β3-adrenergic agonist, CL316,243, on the whole-body metabolism and demonstrated browning resistance of VAT, while SAT was more susceptible to browning conversion. Further, we applied a high-coverage targeted lipidomics approach to determine the effect of CL316,243 on VAT and SAT lipids metabolism when transdifferentiating into BAT. Detection of lipids composition showed that the overall lipids of VAT were dramatically altered adapted to CL316,243 stimulation, including the reduction of triacylglycerol (TAG) and elevation of diacylglycerol (DAG), phosphatidylcholine (PC), phosphatidylethanolamine (PE), lyso-PC (LPC), phosphatidic acid (PA), phosphatidylinositol (PI), cardiolipin, and unexpectedly, glucosylceramide (GluCer) and sphingomyelin (SM). However, CL316,243 just induced a reduction of free fatty acids (FFA) and increase of PC, PE, LPC, and cardiolipin in SAT. The current lipidomics analysis of individual molecular species also revealed that a more significant adaptation of molecular lipid species was present in VAT compared with SAT. Further study uncovered that the lipids remodeling heterogeneity of VAT and SAT may attribute to a more significant alteration of lipids metabolism genes in VAT rather than SAT in response to CL316,243 treatment. In summary, our datasets provide a comprehensive analysis of lipids metabolic heterogeneity between VAT and SAT browning and may provide promising lipid targets to motivate WAT browning.

## 2. Materials and Methods 

### 2.1. Reagents and Antibodies

CL316,243 (C5976) was purchased from Sigma-Aldrich (St. Louis, MO, USA). Antibody against UCP1 was obtained from Abcam (ab10983, Cambridge, UK) and Proteintech (23673-1-AP, Chicago, IL, USA), respectively. Antibody against β Tubulin (TUBB4) was purchased from ZSGB-BIO (Beijing, China).

### 2.2. Animals

The animal protocols were approved by the Institutional Animal Care and Use Committee of the University of Illinois at Chicago. Animal care was given in accordance with institutional guidelines. C57BL/6J male mice were purchased from Jackson Laboratory. The animals were housed in a temperature-controlled and humidity-controlled barrier system with a 12-h light/dark cycle and had free access to standard rodent pellet food and water.

### 2.3. CL316,243 Treatment

Eight-week-old male mice were injected intraperitoneally with CL316,243 (1 mg/kg/d dissolved in phosphate-buffered saline (PBS) ) for 10 days, with PBS-injected mice serving as controls. Bodyweight, 24-h food intake, and water intake were recorded. After the end of 10-day treatment, mice were humanely sacrificed and blood samples were collected. BAT from interscapular region, SAT from inguinal region, and VAT from epididymal region were quickly excised to calculate the fat coefficient with fat weight/body weight, then were immediately frozen in liquid nitrogen for further lipids extraction or fixed in 10% (*wt*/*vol*) buffered formalin for histological diagnosis.

### 2.4. Blood Glucose Determination

After 9-day treatment, fed blood glucose was detected without fasting, and fasting blood glucose was measured after 10-day treatment and overnight fasting by an AccuChek glucometer (FreeStyle Optium Neo, Abbott, USA).

### 2.5. Blood Lipids Detection

After 10-day treatment, blood levels of triglyceride (TG) and free fatty acids (FFA) were detected. The concentration of TG was detected with an automatic analyzer (TOSHIBA Acute TBA-40FR, TOSHIBA CORPORATION, Tokyo, Japan) using the Triglycerides Kit (BioSino Bio-Technology and Science Inc., Beijing, China). The level of FFA was measured according to the manufacturer’s instruction of the Non-Esterified Fatty Acid Kit (BioSino Bio-Technology and Science Inc., Beijing, China).

### 2.6. Dorsal Shell Temperature Detection

At the end of CL316,243 treatment, the dorsal shell temperature was spotted by a thermal imaging camera purchased from FLIR (E8, USA) after the mice were anesthetized.

### 2.7. RNA Isolation and Real-Time PCR

Total RNA was isolated from adipose tissues using Trizol reagent (Invitrogen) and Direct-zol kit (Zymo) following the manufacturer’s recommendations. cDNA was prepared from 2 μg of total RNA according to the instructions of High-Capacity cDNA Reverse Transcription Kit (Invitrogen) with random hexamer primers. The resulting cDNA was diluted ten folds, and a 1.5 μL aliquot was used in a 6 μL PCR reaction (SYBR Green, Bio-Rad) in the presence of primers at a concentration of 300 nM each. PCR reactions were run in triplicate and quantitated using the Applied Biosystems ViiA 7 Real-Time PCR system. Relative amounts of mRNA were normalized to TATA box-binding protein (TBP) expression and expressed as arbitrary units (See Supplemental Table S1 for detailed primer sequences).

### 2.8. Immunoblotting

Total tissue lysates (40 μg) were subjected to SDS-PAGE and immunoblotting was performed as described with anti-UCP1 (Proteintech, 23673-1-AP; 1:1000) or anti-β Tubulin (ZSGB-BIO, TUBB4; 1:1000) antibodies [19].

### 2.9. H&E Staining

Tissues were fixed in 10% (*wt*/*vol*) buffered formalin, and paraffin-embedded sections were subjected to H&E staining. After paraffin embedding, bones were sectioned and stained with hematoxylin and eosin.

### 2.10. Immunohistochemistry Staining

The adipose tissues were fixed in 10% (*wt*/*vol*) buffered formalin and paraffin embedded. 4 μm sections were made and stained with rabbit anti-UCP1 antibody (Abcam, ab10983; 1:500) overnight at 4 ℃. After PBS washing, slides were incubated with goat anti-rabbit IgG (Abcam, 1:2000) for 1 h at room temperature. Finally, staining was completed using DAB Chromogen (DAKO, Glostrup, Denmark). Images were observed under an optical microscope (Olympus BX51) and five randomly selected images per section were digitally captured (400× magnification). 

### 2.11. Lipid Extraction

Lipids were extracted as described before [20]. Briefly, 900 µL of chloroform:methanol (1:2) containing 10% deionized H_2_O were used to inactivate the frozen tissues of VAT and SAT, and then the tissues were homogenized on an automated bead ruptor (OMNI, Kennesaw, GA, USA) using an optimized program and incubated for 1 h at 4 °C with shaking at 1200 rpm. At the end of the incubation, 300 µL of chloroform and 400 µL of deionized H_2_O were added and vortexed. Lower organic phase was collected. A second extraction was carried out via addition of chloroform (500 µL) and 50 µL of 1 M hydrochloric acid. The two organic extracts were pooled into the same tube and dried using a Speed-Vac (Genevac, UK). Samples were stored at −80 °C before high-performance liquid chromatography-tandem mass spectrometry (HPLC-MS/MS) analysis.

### 2.12. Lipidomics Analysis

An ultra-performance liquid chromatography (UPLC) coupled with Sciex QTRAP 6500 Plus was used for UPLC-MS/MS analyses. Individual lipid classes of polar lipids including phospholipids and sphingolipids, as well as free fatty acids, were separated by normal phase HPLC using a Phenomenex Luna 3 µm silica column (150 × 2.0 mm) with mobile phase A (chloroform:methanol:ammonium hydroxide, 89.5:10:0.5) and mobile phase B (chloroform:methanol:ammonium hydroxide:water, 55:39:0.5:5.5). Multiple reaction monitoring (MRM) transitions specific to both head groups and fatty acid chains were set up for quantitative analysis of various phospholipids. Individual lipid species were quantified by comparison with spiked internal standards PC-14:0/14:0, PC34:1-d31, LPC-d4-26:0, PE-14:0/14:0, PE34:1-d31, phosphatidylserines (PS)-14:0/14:0, PS-16:0/18:1-d31, lyso-PS (LPS)-17:1, PA34:1-d31, PA-17:0/17:0, PI34:1-d31, CL-22:1(3)/14:1, ceramides (Cer)-d18:1/17:0, GluCer-d18:1/8:0, galactosylceramide (GalCer)-d18:1/8:0, SM-d18:1/12:0, which were purchased from Avanti Polar Lipids (Alabaster, AL, USA) and LIPIDS MAPS. Dioctanoyl phosphatidylinositol PI-8:0/8:0 (Echelon Biosciences, Inc., Salt Lake City, UT, USA) was used for phosphatidylinositol quantitation. d31-palmitic acid (Sigma-Aldrich, St Louis, MO, USA) and d8-arachidonic acid (Cayman Chemicals, Ann Arbor, MI) were used for quantitation of saturated and polyunsaturated fatty acids, respectively. For all LC/MS analyses, individual peaks were examined and only those above the limit of quantitation and within the linearity range were used for quantitation. The absolute amounts of all qualitative lipid standards were precorrected against quantitative standards before quantitative usage. 

Neutral lipids such as TAG, DAG, and cholesteryl ester (CE) were determined by a modified version of reverse phase HPLC/ESI/MS/MS [21]. HPLC/ESI/MS/MS conditions: Phenomenex Kinetex 2.6 µm-C18 column (i.d. 4.6x100 mm); isocratic mobile phase (chloroform:methanol:0.1M ammonium acetate, 100:100:4); flow rate 150 µL/min for 22 min. Using neutral loss-based MS/MS techniques, TAG species were quantified as relative contents to the spiked d5-TAG 42:0, d5-TAG 48:0, d5-TAG 54:0 internal standards (CDN isotopes), the levels of DAG were calculated using d5-DAG (18:1/18:1) and d5-DAG (16:0/16:0) as internal standards (Avanti Polar Lipids, Alabaster, AL, USA), and CE species were analyzed using d6-CE (CDN isotopes) as internal standard.

In addition, HPLC/APCI/MS/MS was used for the quantitation of free cholesterol (Cho), with corresponding d6-Cho (CDN isotopes) as internal standard.

**Figure 1:**
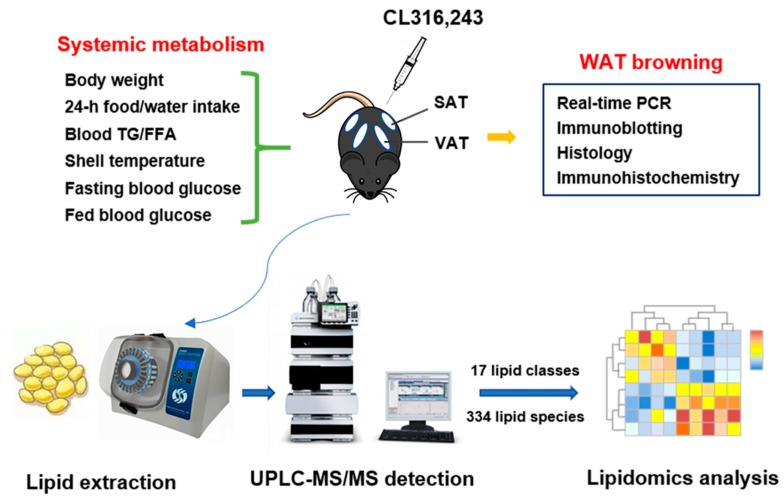
A schematic diagram of experimental design.

A schematic diagram of the experimental design is shown in Figure 1.

### 2.13. Statistical Analysis

Data are presented as mean ± SEM (standard error of mean). Data analysis was performed using Prism software (GraphPad Software Inc.). Statistical analyses were performed by unpaired two-tailed Student’s t-test between two groups after examination of the normal distribution of experimental data by Kolmogorov-Smirnov Test. Independent experiments were performed at least thrice with similar results and studies were performed on five mice per group unless specified. A value of *P* < 0.05 was considered significant.

## 3. Results

### 3.1. Adrenergic Stimulation-Induced Browning Heterogeneity of White Adipose Tissue and Ameliorated Systemic Metabolism

**Figure 2:**
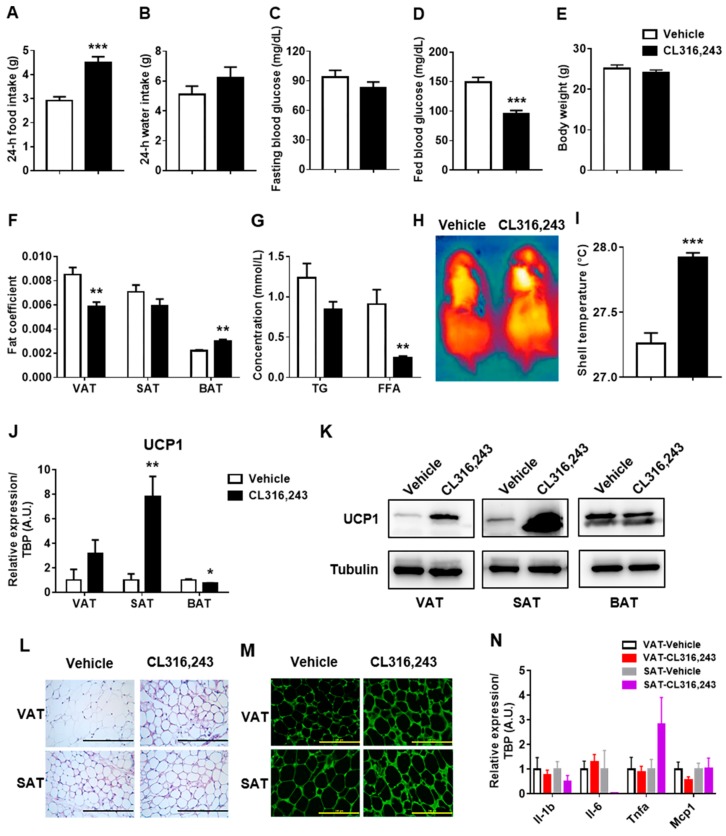
Adrenergic stimulation-induced browning heterogeneity of white adipose tissue and ameliorated systemic metabolism. C57BL/6J mice were injected intraperitoneally with CL316,243 at the dose of 1 mg/kg/d for 10 days and the general parameters were measured (*n* = 5). (**A**) 24-h food intake; (**B**) 24-h water intake; (**C**) Fasting blood glucose; (**D**) Fed blood glucose; (**E**) Body weight; (**F**) Fat coefficient of visceral adipose tissue (VAT), subcutaneous adipose tissue (SAT), and brown adipose tissue (BAT) in vehicle and CL316,243-injected mice; (**G**) The concentration of blood triglyceride (TG) and free fatty acids (FFA); (**H**) The shell temperature was spotted by a thermal imaging camera purchased from FLIR when mice were under anesthesia and (**I**) the temperature was analyzed using FLIR tools. All data are presented as mean ± SEM. ** *P* < 0.01; *** *P* < 0.001 compared with Vehicle group; (**J**) qPCR and (**K**) immunoblotting analysis of UCP1 expression in VAT, SAT, and BAT of mice injected with CL316,243. qPCR data are normalized to TATA box-binding protein (TBP) and presented as mean ± SEM, *n* = 5. * *P* < 0.05; ** *P* < 0.01 compared with Vehicle group; (**L**) H&E staining and (**M**) immunohistochemistry staining of UCP1 in VAT and SAT of mice injected with CL316,243. Scale bar = 200 μm. (**N**) qPCR analysis of interleukin-1 beta (Il-1b), interleukin-6 (Il-6), tumor necrosis factor alpha (Tnfa), and monocyte chemoattractant protein 1 (Mcp1) in VAT and SAT of mice injected with CL316,243. qPCR data are normalized to TBP and presented as mean ± SEM, *n* = 5.

It is well-established that a highly selective β3-adrenergic agonist, CL316,243, plays an important role in regulating energy balance as well as cellular and metabolic remodeling of adipose tissue [22,23,24]. Hence, C57BL/6J mice were intraperitoneally injected with CL316,243 in the present study to explore the effects of adrenergic stimulation on metabolic remodeling. As a result, CL316,243 increased 24-h food intake and slightly elevated 24-h water intake (Figure 2A,B), nevertheless, ameliorated glucose metabolism revealed by a significant reduction of fed blood glucose (Figure 2D) and a mild decline of fasting blood glucose (Figure 2C). Despite no gain or loss in body weight (Figure 2E), dramatic alteration was observed in the distribution of adipose tissue with the reduction of VAT fat coefficient and increase of BAT fat coefficient (Figure 2F) with the reduction of serum TG and FFA levels (Figure 2G). 

To further verify the vital response of adrenergic stimulation on heat production, we spotted dorsal shell temperature by a thermal imaging camera. As expected, we found significant yellow heat signature especially in scapular region as well as shell temperature rise, indicating an augmented thermogenic effect of CL316,243 (Figure 2H,I). Apart from that, we particularly determined browning of VAT and SAT characterized by the unique biomarker of brown fat, UCP1 expression [25]. As a consequence we expected, UCP1 mRNA expression was markedly upregulated by CL316,243 in SAT but mildly upregulated in VAT, and unexpected downregulated in BAT (Figure 2J), which was confirmed by immunoblotting (Figure 2K). Morphological detection by HE staining displayed much more occurrence of multilocular adipocytes in SAT in comparison with VAT (Figure 2L), in combination with the upregulated UCP1 protein expression by immunohistochemistry staining (Figure 2M). Moreover, we observed the trend of attenuated inflammation after CL316,243 stimulation (Figure 2N) in both VAT and SAT, though an upregulated expression of Tnfa was found in SAT, which may be caused by the increased activity of macrophage. Taken together, CL316,243 notably ameliorated the systemic metabolism and induced browning heterogeneity of VAT and SAT with brown remodeling of SAT but browning resistance of VAT.

### 3.2. Total Composition of Lipid Classes in VAT and SAT Altered in Response to CL316,243 Treatment

**Figure 3:**
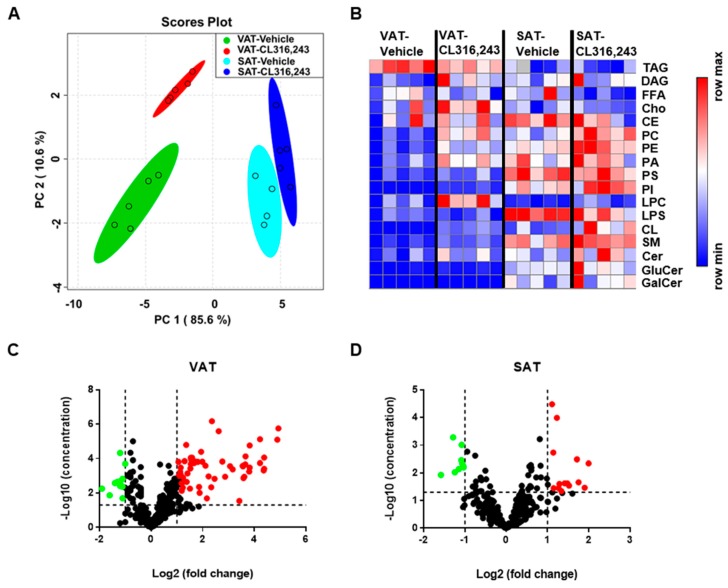
Total composition of lipid classes in VAT and SAT altered in response to CL316,243 treatment. (**A**) Score plot of a multivariate principal component analysis (PCA) of the lipids composition of VAT and SAT from CL316,243-treated C57BL/6J mice; (**B**) Heatmap visualization of the abundance of 17 lipid classes in VAT and SAT from CL316,243-injected mice. In total, 334 lipid species were quantified, and (**C**) 160 molecular species were significantly changed in VAT, while (**D**) 56 molecular species were dramatically altered in SAT.

To deeply understand the browning transdifferentiation heterogeneity induced by β3-adrenergic agonist, we utilized the MRM lipidomics identification for all lipid metabolites of VAT and SAT in response to CL316,243 challenge. Subsequently, comprehensive bioinformatics analysis was performed to characterize the content and composition of total lipids and species, including 17 lipid classes and 334 lipid species. Firstly, a multivariate principal component analysis (PCA) was used to identify different lipids change patterns between VAT and SAT using the concentration data of 17 lipid classes, including TAG, DAG, Cho, CE, FFA, SM, ceramide (Cer), GluCer, galactosylceramide (GalCer), PC, LPC, PS, lyso-PS (LPS), PE, PI, PA, and cardiolipin. Consistent with the phenotype of WAT browning, we observed saliently different lipid profiling between VAT and SAT. Furthermore, a distinct separation of lipid composition exhibited in both VAT and SAT after CL316,243 challenge with more significant changes in VAT (Figure 3A). In addition, heatmap was used to visualize the lipidomics data where the relative abundance detected in adipose tissue is represented with color intensity. The resultant heatmap displayed more dramatic changes of lipid content in majority of individual lipid classes in VAT than in SAT (Figure 3B). More specifically, to gain a more detailed characterization of 17 lipid classes detected, 334 lipid species were quantified, and 160 molecular species significantly changed in VAT illustrated more prominent alteration in lipid species of VAT compared with 56 kinds in SAT (Figure 3C,D). Collectively, adrenergic stimulation-induced browning heterogeneity of VAT and SAT was characterized by the distinctly different lipids content and composition through lipidomics analysis.

### 3.3. Exposure to CL316,243 Differentially Affected the Abundance of Glycerides in VAT and SAT

**Figure 4:**
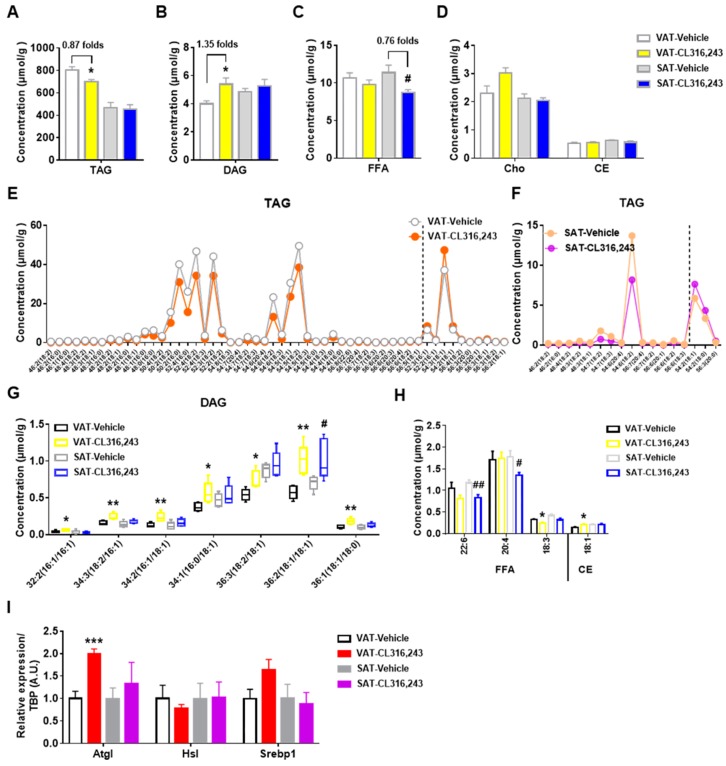
Exposure to CL316,243 differentially affected the abundance of glycerides in VAT and SAT. The total abundance of (**A**) triacylglycerol (TAG), (**B**) diacylglycerol (DAG), (**C**) free fatty acids (FFA), (**D**) cholesterol (Cho), and cholesteryl ester (CE) were detected. The concentration of significantly changed TAG molecular species in (**E**) VAT and (**F**) SAT of mice after CL316,243 injection. The dotted lines separate the raised and lowered species; (**G**) The amount of significantly altered DAG subclasses in VAT and SAT from vehicle and CL316,243-treated mice; (**H**) The abundance of dramatically changed molecular species of FFA and CE in VAT and SAT of mice after CL316,243 treatment. Data are presented as mean ± SEM, *n* = 5. * *P*<0.05; ** *P* < 0.01 compared with VAT Vehicle group. ^#^ *P* < 0.05; ^##^ *P* < 0.01 compared with SAT Vehicle group. (**I**) qPCR analysis of adipose triglyceride lipase (Atgl), hormone-sensitive lipase (Hsl), and sterol regulatory element-binding protein 1 (Srebp1) gene expression in VAT and SAT of mice injected with CL316,243. qPCR data are normalized to TBP and presented as mean ± SEM, *n* = 5. *** *P* < 0.001 compared with VAT Vehicle group.

TAG has been described as an important lipid for excessive energy storage which may cause obesity and associated comorbidities. In the present study, TAG content was measured in VAT and SAT exposed to CL316,243 treatment. As manifested in Figure 4A,B, CL316,243 lowered TAG and elevated DAG level of VAT in line with the reduction of VAT mass (Figure 2F), but did not alter TAG and DAG concentration of SAT. Meanwhile, CL316,243 stimulation also reduced FFA, as by-products of TAG, in both VAT and SAT but without significant differences for VAT (Figure 4C). Additionally, CL316,243 did not affect the abundance of Cho and CE in both VAT and SAT (Figure 4D).

To gain further insight into metabolic profiling, we analyzed the detailed alteration patterns of molecular species in TAG, DAG, FFA, and CE. The majority of TAG species, which were dominated by chain lengths of 46–56 carbons, were significantly decreased in VAT following CL316,243 stimulation (Figure 4E), in agreement with the reduction of total TAG concentration. In contrast, only a few individual TAG species were altered by CL316,243 in SAT, and most of the changed species were overlapped with the species altered in VAT (Figure 4F). The elevated overall DAG content of VAT was possibly due to the increase of seven DAG species with 32, 34, 36 total carbons, while, only one DAG species was augmented in SAT (Figure 4G). Interestingly, although significant reduction was found in total FFA level in SAT, only two FFA species were demonstrated to be statistically lowered in SAT, compared with one species in VAT (Figure 4H). Coincided with unchanged overall concentration, hardly significant change was found in CE molecular species in both VAT and SAT (Figure 4H). In WAT, adipose triglyceride lipase (Atgl) and hormone-sensitive lipase (Hsl) are responsible for hydrolysis of about 95% TAG [26]. Sterol regulatory element-binding protein 1 (Srebp1) is a critical transcriptional regulator of cholesterol and fatty acid metabolism. Further study showed that Atgl mRNA expression of VAT was dramatically upregulated by CL316,243 injection without changes in SAT, which was consistent with the lipidomics results of glycerides. Whereas, Hsl and Srebp1 mRNA expression were not significantly altered in both VAT and SAT (Figure 4I). Overall, VAT experienced different glycerides metabolic remodeling from SAT in response to adrenergic browning induction.

### 3.4. Adrenergic Stimulation Boosted the Abundance of More Glycerophospholipids Species in VAT Than in SAT 

**Figure 5:**
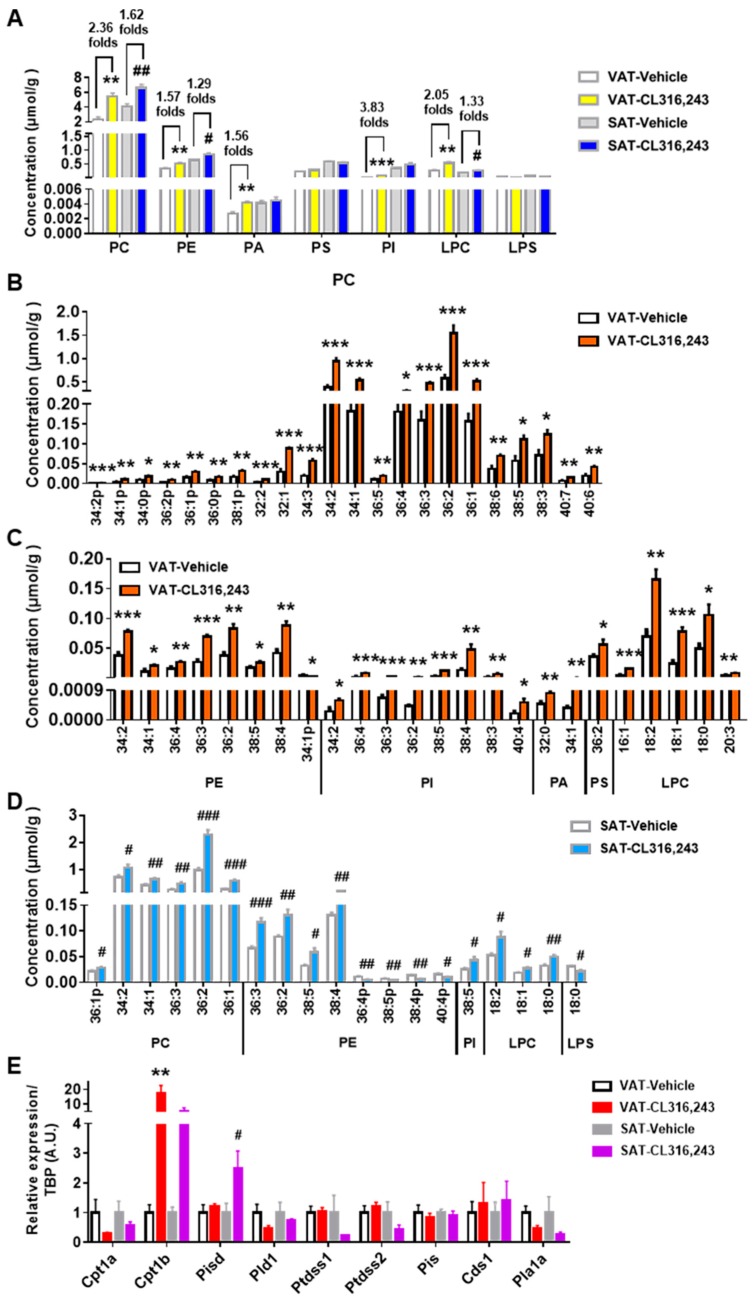
Adrenergic stimulation boosted the abundance of more glycerophospholipids species in VAT than in SAT. (**A**) The overall concentration of classical glycerophospholipids was determined. The abundance of significantly varied (**B**) phosphatidylcholine (PC), and (**C**) phosphatidylethanolamine (PE), phosphatidylinositol (PI), phosphatidic acid (PA), phosphatidylserines (PS), lyso-PC (LPC) species in VAT; (**D**) The obviously changed molecular species of PC, PE, PI, LPC, and lyso-PS (LPS) in SAT. Data are presented as mean ± SEM, *n* = 5. * *P* < 0.05; ** *P* < 0.01; *** *P* < 0.001 compared with VAT Vehicle group. ^#^ *P* < 0.05; ^##^ *P* < 0.01; ^###^ *P* < 0.001 compared with SAT Vehicle group. (**E**) qPCR analysis of carnitine palmitoyltransferase 1a (Cpt1a), carnitine palmitoyltransferase 1b (Cpt1b), phosphatidylserine decarboxylase (Pisd), phospholipase D1 (Pld1), phosphatidylserine synthase 1 (Ptdss1), phosphatidylserine synthase 2 (Ptdss2), phosphatidylinositol synthase (Pis), CDP-diacylglycerol synthase 1 (Cds1), and phospholipase A1 member A (Pla1a) gene expression in VAT and SAT of mice injected with CL316,243. qPCR data are normalized to TBP and presented as mean ± SEM, *n* = 5. ** *P* < 0.01 compared with VAT Vehicle group. ^#^ *P* < 0.05 compared with SAT Vehicle group.

Glycerophospholipids serve as major constituents of cellular and organellar membranes and participate in diverse biological processes. To elucidate the change patterns of glycerophospholipids in VAT and SAT induced by browning stimulation, seven main glycerophospholipids classes were analyzed. All in all, our analysis revealed that PC was the predominate class in both VAT and SAT, followed by PE, PS, and LPC. CL316,243 elevated the total amount of PC, PE, PA, PI, LPC in VAT and PC, PE, LPC in SAT without any change in the overall abundance of PS and LPS. More importantly, higher fold changes in elevated lipid classes were found in VAT than in SAT (Figure 5A).

To gain a more informative perspective of glycerophospholipids profiling, molecular species of each glycerophospholipids class were quantified. In VAT, 22 out of 34 PC species were markedly elevated, including highly abundant species with side chains of 34:2, 34:1, 36:4, 36:3, 36:2, and 36:1 (Figure 5B). PE species containing 34:2, 34:1, 36:4, 36:3, 36:2, 38:5, or 38:4 chains were also increased by CL316,243 apart from the reduction of minor abundant PE species 34:1p (Figure 5C). In addition, all 8 PI species and 2 out of 5 PA species detected in this study were significantly boosted (Figure 5C). For PS and LPS species, just PS 36:2 was augmented without alteration in LPS species (Figure 5C). Moreover, there was obvious elevation of 3 highly abundant LPC species containing 18:2, 18:1, or 18:0 chains and 2 minor abundant LPC species containing 16:1 or 20:3 chains (Figure 5C).

Interestingly, CL316,243 elevated several PC species of SAT including the highly abundant 34:2, 34:1, 36:3, 36:2, 36:1 species and less abundant 36:1p species (Figure 5D) with lower elevation fold changes than VAT. In addition, there was an overlap in PE species of VAT and SAT that exhibited increase in response to CL316,243, including PE 36:3, 36:2, 38:5, and 38:4, however, PE 36:4p, 38:5p, 38:4p, and 40:4p were uniquely reduced in SAT (Figure 5D). Furthermore, only PI 38:5 and LPS 18:0 were altered by CL316,243 without any alteration in PA and PS in SAT (Figure 5D). The increase of overall LPC concentration in SAT was driven by the elevation of three highly abundant LPC species with side chains of 18:2, 18:1, or 18:0 (Figure 5D), in accordance with VAT. To explore the underlying mechanism of the findings, we examined the expression of glycerophospholipids metabolism genes. It showed that carnitine palmitoyltransferase 1b (Cpt1b), responsible for the synthesis of PC, was more markedly upregulated in VAT than in SAT, whereas, phosphatidylserine decarboxylase (Pisd), which converts PS to PE, was significantly upregulated in SAT instead of VAT. Interestingly, no alteration of phosphatidylserine synthase 1 (Ptdss1) and phosphatidylserine synthase 2 (Ptdss2) was found in VAT after adrenergic stimulation but a downregulated trend was observed in SAT as PS Synthase. However, we did not see obvious changes for carnitine palmitoyltransferase 1a (Cpt1a), phospholipase D1 (Pld1), phosphatidylinositol synthase (Pis), CDP-diacylglycerol synthase 1 (Cds1), and phospholipase A1 member A (Pla1a) expression (Figure 5E). Nonetheless, to confirm the glycerophospholipids species as the biomarkers for WAT browning, further investigations are still needed. 

### 3.5. CL316,243 Increased Sphingolipids in VAT without Changes in SAT 

**Figure 6:**
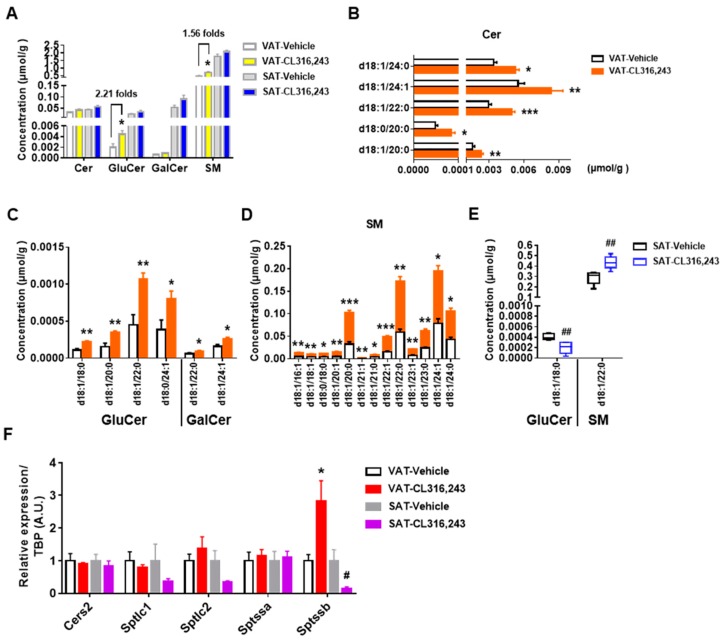
CL316,243 increased sphingolipids in VAT without changes in SAT. (**A**) The total concentration of sphingolipids in VAT and SAT were quantified. Concentration of (**B**) ceramide (Cer), (**C**) glucosylceramide (GluCer), galactosylceramide (GalCer), and (**D**) sphingomyelin (SM) species significantly changed in VAT after 10 days of CL316,243 injection; (**E**) The molecular species of GluCer and SM changed in VAT by CL316,243. All data are presented as mean ± SEM, *n* = 5. * *P* < 0.05; ** *P* < 0.01; *** *P* < 0.001 compared with VAT Vehicle group. ^##^ *P* < 0.01 compared with SAT Vehicle group. (**F**) qPCR analysis of ceramide synthase 2 (Cers2), serine palmitoyltransferase long chain base subunit 1 (Sptlc1), serine palmitoyltransferase long chain base subunit 2 (Sptlc2), serine palmitoyltransferase small subunit A (Sptssa) and serine palmitoyltransferase small subunit B (Sptssb) gene expression in VAT and SAT of mice injected with CL316,243. qPCR data are normalized to TBP and presented as mean ± SEM, *n* = 5. * *P* < 0.05 compared with VAT Vehicle group. ^#^ *P* < 0.05 compared with SAT Vehicle group.

As relatively minor constituents of lipids, sphingolipids can be broadly divided into ceramide, sphingomyelin, and glycosphingolipid. Ceramide is the precursor for most sphingolipids and is synthesized from FFA and serine [27]. In our current study, CL316,243 exhibited a tendency to increase sphingolipids levels, especially for GluCer and SM in VAT without significant changes in the abundance of SAT sphingolipids (Figure 6A).

To determine the species contributing to the elevation, 52 sphingolipids species were identified respectively. Although there were no significant differences in total concentrations of Cer and GalCer in VAT, significant augment in the content of Cer d18:1/20:0, d18:0/20:0, d18:1/22:0, d18:1/24:1, d18:1/24:0 and GalCer d18:1/22:0, d18:1/24:1 were detected (Figure 6B,C). Four kinds of GluCer species in VAT were increased, including GluCer d18:1/18:0, d18:1/20:0, d18:1/22:0, and d18:0/24:1 (Figure 6C). Additionally, 13 specific molecular species were responsible for the elevated level of SM in VAT (Figure 6D). However, only the reduction of GluCer d18:1/18:0 and the increase of SM d18:1/22:0 were observed in SAT (Figure 6E). We then detected the gene expression related to sphingolipids metabolism and found the significant upregulation of serine palmitoyltransferase small subunit B (Sptssb) and the upregulated trend of serine palmitoyltransferase long chain base subunit 2 (Sptlc2) but the downregulated trend of serine palmitoyltransferase long chain base subunit 1 (Sptlc1) in VAT. However, Sptssb, Sptlc1 and Sptlc2 all showed a downregulated tendency in SAT. Besides, no significant alteration of ceramide synthase 2 (Cers2) and serine palmitoyltransferase small subunit A (Sptssa) were found in both VAT and SAT (Figure 6F), which partially confirmed our lipidomics results. Collectively, our findings were not consistent with the previous study probably due to the different exposure conditions which might induce the remarkable alteration in adipose tissue [17]. More importantly, the differences in sphingolipids metabolism between VAT and SAT may be the clue to address the browning heterogeneity in future.

### 3.6. Cardiolipin was Elevated by CL316,243 in both VAT and SAT 

**Figure 7:**
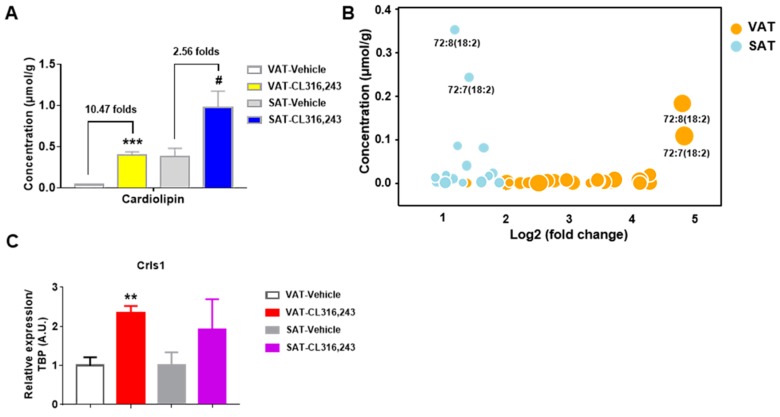
Cardiolipin was elevated by CL316,243 in both VAT and SAT. (**A**) The total content of cardiolipin was significantly altered in both VAT and SAT in response to CL316,243 treatment. In 35 species detected, (**B**) 27 molecular species were obviously changed in VAT, and 15 species were greatly altered in SAT. Dramatically changed particular species of cardiolipin are illustrated as circles. Size of circle indicates level of significance. All data are presented as mean ± SEM, *n* = 5. *** *P* < 0.001 compared with VAT Vehicle group. ^#^ *P* < 0.05 compared with SAT Vehicle group. (**C**) qPCR analysis of cardiolipin synthase 1 (Crls1) gene expression in VAT and SAT of mice injected with CL316,243. qPCR data are normalized to TBP and presented as mean ± SEM, *n*=5. ** *P* < 0.01 compared with VAT Vehicle group.

As the signature phospholipid of mitochondrial membranes, cardiolipin is closely associated with mitochondrial function and structural integrity [28,29,30,31]. To determine the mitochondrial changes of WAT under CL316,243 challenge, the abundance of total cardiolipin and 35 species were detected. As showed in Figure 7A, we observed the significant elevation of cardiolipin content in both VAT and SAT with 10.47 folds significant increase for VAT and 2.56 folds for SAT. Analysis of molecular species in cardiolipin indicated a rise tendency for almost all species, among these, 27 cardiolipin species in VAT were statistically increased, compared with 15 species for SAT (Table 1). The increase in cardiolipin abundance probably revealed the higher mitochondrial content in VAT and SAT in response to CL316,243. Our results also identified that 18:2 and 16:1 acyl groups were the most dominated acyl side chains in cardiolipin species in both VAT and SAT (Table 1). The findings were consistent with previous observations of mitochondrial cardiolipin composition in diverse eukaryotes [32].

More interestingly, C72:8 (18:2) and C72:7 (18:2) are the most abundant cardiolipin species in VAT and SAT after CL316,243 treatment. In VAT, C72:8 (18:2) and C72:7 (18:2) dramatically enhanced by 29.73 and 30.46 folds, and constituted 45.76% and 27.17% of total cardiolipin, respectively. However, C72:8 (18:2) and C72:7 (18:2) increased by 2.44 and 2.87 folds, and made up 36.22% and 25.01% of overall cardiolipin in SAT (Figure 7B). This composition was quite similar as the cardiolipin profiling in highly oxidative tissues, such as cardiac and skeletal muscles [33]. Cardiolipin synthase 1 (Crls1) catalyzes the synthesis of cardiolipin. Our study showed that Crls1 mRNA expression was more significantly upregulated in VAT than in SAT, in agreement with the more folds increase of cardiolipin content in VAT (Figure 7C). Taken together, we identified some species of cardiolipin as the important potential biomarkers of WAT browning, especially C72:8 (18:2) and C72:7 (18:2).

## 4. Discussion

Obesity is a major risk factor for the development of prediabetes and type 2 diabetes mellitus (DM) [34]. WAT browning has been proposed as a promising therapeutic approach against obesity; however, VAT, the most pathogenic adipose tissue, is less susceptible to browning than SAT evidenced by our current study. Therefore, illustrating the possible mechanisms involved in this process is extremely important. In the present study, UPLC-MS/MS was utilized to analyze the lipidomics alterations of VAT and SAT in browning transdifferentiation induced by β3-adrenergic agonist. We clearly demonstrated that compared with SAT, VAT has undergone a more significant metabolic remodeling in glycerides, glycerophospholipids, sphingolipids, and cardiolipin driven by species-specific alteration, which indicated the browning heterogeneity between VAT and SAT though VAT is resistant to browning. 

Visceral fat accumulation is associated with serum triglyceride levels. Moreover, VAT adipocytes are also more sensitive to lipolysis and more prone to develop insulin resistance than SAT adipocytes [35,36]. Activation of the adrenergic receptor could produce increased lipolysis and elevated fatty acids in plasma [37]. Not surprisingly, we observed that the reduction of total TAG in VAT was accompanied by an elevation of overall DAG, which attributed to species-specific adaptation to adrenergic stimulation. Conversely, the abundance of total SAT glycerides remained unchanged though there were significant alteration of several molecular species of TAG and DAG. Previous study has established a heterogeneous TAG signature which predicted that lower carbon number and double bond content of TAG, including TAG 44:1, 46:1, 48:0, 48:1, 50:0, and 52:1, are associated with an elevated risk of type 2 diabetes [38]. Consistently, we found that the content of TAG 46:1, 48:0, and 48:1 was markedly lowered in VAT, coupled with decreased blood glucose, indicating that the downregulation of TAG content in VAT might contribute more to the improved glucose metabolism. It has been proposed that TAG metabolism is important for activation of UCP1 and acquisition of a multilocular phenotype in adipose tissue, ultimately leading to heat generation and browning of WAT [39,40]. Therefore, the alteration of TAG in the present study might be a motivation as well as a reflection of VAT and SAT browning adapted to β3-adrenergic stimulation. Besides, as a more dramatic depletion in TAG level was observed in VAT rather than SAT, the lipolysis of TAG in VAT might be more likely responsible for the upregulated non-shivering thermogenesis after CL316,243 treatment. More specifically, detection of TAG metaboliam genes showed that Atgl may be responsible for the reduction of TAG in VAT.

Glycerophospholipids are major constituents of cellular and organellar membranes which participate in diverse biological processes. Among them, PC, the most abundant mitochondrial membrane phospholipid, is a critical constituent of both inner and outer mitochondrial membranes. In addition, PE accounts for a large portion in the inner mitochondrial membrane of mammalian cells. PC and PE are representative of nearly 80% of total mitochondrial phospholipids [41]. Our lipidomics analysis found that the total concentration as well as the content of majority of individual molecular species were upregulated in PC, PE, and LPC in both VAT and SAT to accommodate adrenergic stimulation, and species-specific elevation was more pronounced in VAT than in SAT. Since PC and PE have shown multiple physiological functions in metabolic diseases, particularly alleviating obesity and its related comorbidities [42,43,44], therefore, the elevation of PC and PE might contribute to lipid supply for mitochondria biogenesis as well as heat production in WAT, further ameliorate lipid metabolism. However, the association of individual PC and PE species with VAT and SAT browning is not yet well characterized.

PA is an essential precursor for the biosynthesis of most phospholipids and glycerolipids [45]. PI is enriched in the outer mitochondrial membrane of mammalian cells and serves as a substrate for phosphoinositides and inositol-containing sphingolipids synthesis [41,46]. Interestingly, in response to CL316,243, the overall abundance of PI and PA was uniquely increased in VAT but not in SAT, demonstrating a much more active lipids metabolism in VAT compared with SAT. Inconsistent with adrenergic stimulation, no significant change is found in lipids metabolism of SAT after cold exposure [15]. Meanwhile, three-week exercise training significantly decreased SAT mass with concomitant reduction of individual molecular species of PA, PC, PE, and PS [14]. This discrepancy might be probably due to the different induction or stimulation. WAT browning needs the acquisition of more mitochondria and transdifferentiation to a multilocular phenotype, thus more membrane structure and compartmentalization will be synthesized. However, cold exposure or exercise may alter not only WAT but also non-adipose tissue metabolism, which taken together does not change or even decreases glycerophospholipids abundance. It still needs to elucidate whether the increased glycerophospholipids are hallmarks of WAT browning.

Sphingolipids have been regarded as a principal lipid class connecting obesity to its complications, such as type 2 diabetes and cardiovascular disease. As a precursor of all complex sphingolipids, ceramide is composed of a sphingosine backbone coupled to acyl-chains containing 14–30 carbons. Recently, Chaurasia et al. have reported that cold exposure and CL316,243 treatment decreased the content of ceramide in SAT [17]. Quite unexpectedly, our findings demonstrated that CL316,243 increased the abundance of some individual molecular species of Cer, GluCer, GalCer, and SM in VAT, though significant elevation of total concentration was just found in GluCer and SM. Whereas, almost none change of sphingolipids were observed in SAT. The different duration of CL316,243 treatment between 3 days of Chaurasia and 10 days of our current study might be a key factor for moderating sphingolipids metabolism. In addition, studies have deciphered the connection between pathogenicity of ceramide with its side chain length and saturability. C16:0- and C18:0-Cer have been revealed as critical mediator of obesity-related metabolic diseases [47,48,49], whereas very-long-chain Cer, such as C24:0 or C24:1, may have beneficial effects on metabolic disorders [50]. Regarding the alteration of VAT, we assumed that ceramide species elevated in our study might be just a revelation of activated lipids metabolism in response to CL316,243 stimulation, which are less pathogenic. Whereas, further investigation needs to evaluate whether the effects of ceramide are beneficial to metabolism or not.

SM is the most abundant sphingolipids and sphingosine (i.e., d18:1) is the most common long-chain base present in SM [51]. The high levels of SM species containing saturated acyl chains (C18:0, C20:0, C22:0, and C24:0) have been considered to be tightly linked to metabolic diseases, including obesity and insulin resistance [52]. Our analysis demonstrated that CL316,243-induced sphingolipids elevation was only present in VAT but not in SAT, including certain species of Cer, GluCer, GalCer, and SM. In view of the possible pathogenic effects of sphingolipids, further study is required to identify whether the increase represents browning remodeling or browning resistance of VAT.

As a specific phospholipid of mitochondrial membranes, cardiolipin extensively interacts with mitochondrial proteins, including ADP/ATP carrier, complex I, III, and V, thus has been considered as an integral part of oxidative phosphorylation complexes to regulate mitochondrial function in particular oxidative phosphorylation [29,41]. It has been demonstrated that cold exposure induced cardiolipin increase in BAT and SAT, as a biomarker of thermogenesis and browning [16]. However, exercise induced cardiolipin reduction in BAT but without any change in SAT [14]. In accordance with cold exposure findings, our lipidomics analysis revealed that cardiolipin markedly increased after adrenergic stimulation with 10.47 folds increase in VAT and 2.56 folds increase in SAT, indicating that dramatical mitochondrial biogenesis occurred in WAT response to adrenergic stimulation, especially in VAT. More interestingly, it has been reported that the biological function of cardiolipin was mainly ascribed to the 18:2 fatty acyl. Cold exposure increases the content of cardiolipin 18:2 acyl in BAT, and depletion of 18:2 acyl has been observed in various diseases [53]. Indeed, we observed that cardiolipin highly abundant C72:8 (18:2) and C72:7 (18:2) species were increased in VAT and SAT by CL316,243 with more elevation in VAT than in SAT. Therefore, we identified cardiolipin, especially C72:8 (18:2) and C72:7 (18:2) species as the potential biomarkers of WAT browning under adrenergic responsiveness, in consistence with Tseng’s findings [16]. Furthermore, our study revealed that Crls1 may contribute to the elevated cardiolipin metabolism.

In summary, our current study mainly observed the lipid profiling of VAT and SAT under adrenergic stimulation of CL316,243 for 10 days and found lipids remodeling heterogeneity of VAT and SAT which may attribute to a more significant alteration of lipids metabolism genes in VAT rather than SAT. Nonetheless, further studies are needed to identify more biologically active lipid species to elucidate the browning heterogeneity between VAT and SAT, especially VAT browning resistance under different browning conditions and promote WAT browning.

## 5. Conclusions

The present study has demonstrated the browning heterogeneity of WAT induced by adrenergic stimulation by lipidomics signature. Though VAT is not as susceptible to browning as SAT, lipidomics analysis reveals a more significant alteration of lipids remodeling of VAT than SAT in response to CL316,243 treatment. Our study provides lipidomics insight into VAT and SAT browning heterogeneity which may provide promising lipid targets for promoting WAT browning. 

## Figures and Tables

**Table 1:** Changes of cardiolipin species in VAT and SAT of mice under CL316,243 stimulation.

Lipid Species	VAT			SAT		
	Fold Changes	*P* Value		Fold Changes	*P* Value	
68:6(16:1)	20.52699194	0.000475	***	1.766938316	0.079321	
68:5(16:1)	13.97259484	0.000564	***	2.008876544	0.026587	#
70:8(16:1)	20.77862561	8.64 × 10^−05^	***	2.005579532	0.10948	
70:7(16:1)	11.95160231	0.001351	**	1.979040735	0.016	#
70:6(16:1)	14.10403798	0.000329	***	2.406633843	0.035636	#
70:6(18:2)	20.88860213	0.000393	***	2.789402423	0.023778	#
70:5(16:1)	12.5429712	0.000212	***	2.560187318	0.053366	
70:5(18:2)	14.13490498	1.74 × 10^−05^	***	3.72660512	0.034428	#
70:4(16:1)	12.17132084	0.001126	**	3.984337045	0.004524	##
70:4(18:2)	12.61352092	7.52 × 10^−05^	***	3.035547901	0.055842	
72:10(18:2)	5.013887345	0.004586	**	1.243596576	0.601292	
72:9(16:1)	10.73074822	0.028529	*	0.678223578	0.437706	
72:9(18:2)	18.59530648	7.67 × 10^−06^	***	2.16986299	0.050667	
72:8(18:2)	29.73292901	8.01 × 10^−06^	***	2.441405669	0.025102	#
72:8(20:3)	3.598494043	0.059739		1.292607526	0.554198	
72:7(16:1)	18.62111688	0.000183	***	2.667047606	0.023761	#
72:7(18:2)	30.45759488	1.79 × 10^−06^	***	2.86873778	0.02882	#
72:6(16:1)	2.799529192	0.046693	*	1.369963332	0.485846	
72:6(18:2)	7.291839788	0.001115	**	3.364376204	0.021878	#
72:5(18:2)	—	—		3.561477455	0.024244	#
74:11(16:1)	2.745578422	0.053239		2.345654479	0.084162	
74:11(18:2)	2.139752181	0.134496		1.248345849	0.627876	
74:10(18:2)	11.78634294	0.000284	***	1.790233115	0.080245	
74:10(20:4)	8.968951403	0.000418	***	1.193409387	0.752919	
74:9(18:2)	11.71784353	0.000263	***	1.512566398	0.23822	
74:9(20:3)	5.513657707	0.00148	**	1.035975783	0.909981	
74:8(18:2)	8.34535855	0.000278	***	1.663407978	0.171988	
74:8(20:3)	3.029370298	0.078375		1.115185366	0.774192	
76:13(18:2)	6.755548969	0.000149	***	2.524297408	0.045155	#
76:12(16:1)	6.154661722	2.55 × 10^−06^	***	3.278898397	0.003226	##
76:12(18:2)	4.323385873	0.000256	***	2.217419145	0.035696	#
76:10(18:2)	4.457581419	0.020536	*	1.521167675	0.226874	
76:10(20:3)	0.449088362	0.556864		0.778169257	0.503334	
76:9(18:2)	2.536667231	0.07915		0.752584244	0.496683	
78:13(18:2)	2.141719426	0.12503		2.206003273	0.001864	##

Fold changes represented the concentration of CL316,243 treated group divided by the concentration of Vehicle group. Data are presented as mean ± SEM, *n* = 5. * *P* < 0.05; ** *P* < 0.01; *** *P* < 0.001 compared with VAT Vehicle group; ^#^ *P* < 0.05; ^##^ *P* < 0.01; ^###^ *P* < 0.001 compared with SAT Vehicle group.

## Supplementary Materials

The following are available online at https://www.mdpi.com/2218-273X/9/9/444/s1. Table S1: Primer sequences for Real time RT-PCR.
