# Supplementary material for: Lipid Profiling Reveals Browning Heterogeneity of White Adipose Tissue by Β3-Adrenergic Stimulation
Source: Biomolecules. 2019 Sep 3;9(9):444. doi: 10.3390/biom9090444 (PMC6770315; doi:10.3390/biom9090444)
Supplement: Supplementary file 1 [file biomolecules-09-00444-s001.pdf]

SUPPLEMENTAL INFORMATION:

Lipid profiling reveals browning heterogeneity of white adipose tissue by β3-adrenergic stimulation

Ping He [1], Biyu Hou [1], Yanliang Li [2], Chunyang Xu [1], Peng Ma [1], Sin Man Lam [3], Victoria Gil [2], Xinyu Yang [4], Xiuying Yang [1], Li Zhang [1], Guanghou Shui [3], Junke Song [1], Guifen Qiang [1,*], Chong Wee Liew [2,*] and Guanhua Du [1,*]

[1] State Key Laboratory of Bioactive Substances and Functions of Natural Medicines, Institute of Materia Medica, Chinese Academy of Medical Sciences and Peking Union Medical College and Beijing Key Laboratory of Drug Target and Screening Research, Beijing 100050, China

[2] Department of Physiology and Biophysics, College of Medicine, University of Illinois at Chicago, Chicago, 60612 Illinois, USA

[3] State Key Laboratory of Molecular Developmental Biology, Institute of Genetics and Developmental Biology, Chinese Academy of Sciences, Beijing 100101, China

[4] College of Pharmacy, Guangdong Medical University, Dongguan 523808, China

* Correspondence: qianggf@imm.ac.cn (G.Q.); cwliew@uic.edu (C.W.L.); dugh@imm.ac.cn (G.D.)

Supplemental Table S1. Primer sequences for Real time RT-PCR.

Primer	Sequences
Ucp1	Forward: 5'- CTGCCAGGACAGTACCCAAG -3'
	Reverse: 5'- TCAGCTGTTCAAAGCACACA -3'
Il-1b	Forward: 5'- TGGAGAGTGTGGATCCCAAGCAAT -3'
	Reverse: 5'- TGTCCTGACCACTGTTGTTTCCCA -3'
Il-6	Forward: 5'- ATCCAGTTGCCTTCTTGGGACTGA -3'
	Reverse: 5'- TAAGCCTCCGACTTGTGAAGTGGT -3'
Tnfa	Forward: 5'- GCCTCTTCTCATTCCTGCTTGT -3'
	Reverse: 5'- GGCCATTTGGGAACTTCTCAT -3'
Mcp1	Forward: 5'- CCACTCACCTGCTGCTACTCAT -3'
	Reverse: 5'- TGGTGATCCTCTTGTAGCTCTCC -3'
Atgl	Forward: 5'- TAGCTAACAGTTGGGCTTCAC -3'
	Reverse: 5'- CAGAGAGAACAGAGCAGCTTAC -3'
Hsl	Forward: 5'- ACGGATACCGTAGTTTGGTGC -3'
	Reverse: 5'- TCCAGAAGTGCACATCCAGGT -3'
Srebp1	Forward: 5'- CAAGGCCATCGACTACATCCG -3'
	Reverse: 5'- CACCACTTCGGGTTTCATGC -3'
Cpt1a	Forward: 5'- CTCCGCCTGAGCCATGAAG -3'
	Reverse: 5'- CACCAGTGATGATGCCATTCT -3'

Cpt1b	Forward: 5'- TGAGACCAGTCTTAGCCTCTAC -3'
	Reverse: 5'- GGCCATTCTTGCAGGAGATAA -3'
Pisd	Forward: 5'- TCTGGGGACCTTACAGAAATTGC -3'
	Reverse: 5'- GGCACAGATTTATACAGGGACAC -3'
Pld1	Forward: 5'- CTGCCCCGTTCATCAGAAAAC -3'
	Reverse: 5'- GCCCTTTGGTCCCAGATCAT -3'
Ptdss1	Forward: 5'- GCAGGACTCTGAGCAAGGATG -3'
	Reverse: 5'- GGCGAAGTACATGAGGCTGAT -3'
Ptdss2	Forward: 5'- GGATTGCCTTTCAGTTCACGC -3'
	Reverse: 5'- AGGTAGAAGGTGTTCAGCTCTG -3'
Pis	Forward: 5'- TCCTGTTCGTGCCTAACCTTA -3'
	Reverse: 5'- AGGAGTCCGCTGAGTAGATAGA -3'
Cds1	Forward: 5'- GGTGACCACGAAACCGAGAG -3'
	Reverse: 5'- CCGCGAATCCACCAGTTCT -3'
Pla1a	Forward: 5'- GGTTGTGGGGACCACTTTTATG -3'
	Reverse: 5'- CACCTTGAGGTTGGTGCCT -3'
Cers2	Forward: 5'- TATGACTACTTCTGGTGGGAACG -3'
	Reverse: 5'- GTATCGAATGACGAGAAAGAGCA -3'
Sptlc1	Forward: 5'- ACGAGGCTCCAGCATACCAT -3'
	Reverse: 5'- TCAGAACGCTCCTGCAACTTG -3'

Sptlc2	Forward: 5'- CATGCCCTGGATCTGTTATCTT -3'
	Reverse: 5'- AGCCTGTTCACCCATTTCTAC -3'
Sptssa	Forward: 5'- ACCGTGTTCAATTCGATGCTG -3'
	Reverse: 5'- GGGGCATGAAGACGTAGCC -3'
Sptssb	Forward: 5'- CGTGAAGGAGTATTTTGCCTGG -3'
	Reverse: 5'- GCCACAATGGTCAGTATGATGGT -3'
Crls1	Forward: 5'- GTGTTGCACAGCATTCACTAC -3'
	Reverse: 5'- GTGCTTCTCATTTGCCCTTTATC -3'
Tbp	Forward:5'- ACCCTTCACCAATGACTCCTATG -3'
	Reverse: 5'- ATGATGACTGCAGCAAATCGC -3'
